# Myofibroblasts are increased in the lung parenchyma in asthma

**DOI:** 10.1371/journal.pone.0182378

**Published:** 2017-08-07

**Authors:** Stacey R. Boser, Thais Mauad, Bianca Bergamo de Araújo-Paulino, Ian Mitchell, Grishma Shrestha, Andrea Chiu, John Butt, Margaret M. Kelly, Elia Caldini, Alan James, Francis H. Y. Green

**Affiliations:** 1 Airway Inflammation Group, Snyder Institute of Chronic Diseases, Faculty of Medicine, University of Calgary, Calgary, Alberta, Canada; 2 Department of Pathology, School of Medicine, University of São Paulo, São Paulo, Brazil; 3 Pathfinder Forum, Forensic Pathology, Vancouver, British Columbia, Canada; 4 Department of Pulmonary Physiology and Sleep Medicine, Sir Charles Gairdner Hospital, Perth, Western Australia, Australia; National and Kapodistrian University of Athens, GREECE

## Abstract

**Background:**

Increased airway smooth muscle is observed in large and small airways in asthma. Semi-quantitative estimates suggest that cells containing alpha smooth muscle actin (α-SMA) are also increased in the lung parenchyma. This study quantified and characterized α-SMA positive cells (α-SMA+) in the lung parenchyma of non-asthmatic and asthmatic individuals.

**Methods:**

Post-mortem sections of peripheral lung from cases of fatal asthma (FA), persons with asthma dying of non-respiratory causes (NFA) and non-asthma control subjects (NAC) were stained for α-SMA, quantified using point-counting and normalised to alveolar basement membrane length and interstitial area.

**Results:**

α-SMA+ fractional area was increased in alveolar parenchyma in both FA (14.7 ± 2.8% of tissue area) and NFA (13.0 ± 1.2%), compared with NAC (7.4 ± 2.4%), p < 0.05 The difference was greater in upper lobes compared with lower lobes (p < 0.01) in both asthma groups. Similar changes were observed in alveolar ducts and alveolar walls. The electron microscopic features of the α-SMA+ cells were characteristic of myofibroblasts.

**Conclusions:**

We conclude that in asthma there is a marked increase in α-SMA+ myofibroblasts in the lung parenchyma. The physiologic consequences of this increase are unknown.

## Introduction

Asthma is characterized by inflammatory and structural alterations in central and distal lung compartments [1, 2]. Individuals with asthma show differences in tissue matrix components and inflammatory cell patterns in the distal lung compared with non-asthmatic individuals and such alterations are thought to be linked to disease and lack of control [2–5].

Among the most prominent and consistent structural alterations in asthma, clearly linked to functional outcomes, are hypertrophy and hyperplasia of airway smooth muscle (ASM) cells seen in large and small airways [6]. Changes in ASM appear early in the natural history of asthma and relate to severity but not duration of disease [7]. The causes of the increased ASM mass and the effects of current therapy are not known. Contractile cells, characterized by bundles of intracellular actin filaments, are seen in the lung parenchyma [3, 8, 9]. In normal lungs, alpha-smooth muscle actin containing cells (α-SMA+) are observed in airways, bronchi, bronchioles, and blood vessels[8]. α-SMA+ cells distal to respiratory bronchioles have also been identified and consist of cells within and around arterioles and venules and myofibroblasts located in alveolar ducts and in the alveolar wall interstitium [8]. These cells in the lung parenchyma are thought to play an important role in matching ventilation to perfusion [10, 11], in the regulation of lung elasticity [9, 11] and in fibrosis and repair [3, 12]. Contractility of alveolar units has been demonstrated in studies using peripheral lung strips in response to contractile agonists, in the absence of bronchioles [10]. Thus changes in the structural integrity of the lung parenchyma may play an important role in asthma and contribute to airway dysfunction [8].

Previous work by our group has shown that there is both hypertrophy and hyperplasia of ASM cells [6] and increased myoepithelial cells in the mucous glands from individuals with asthma [13]. For this study our general hypothesis was that remodelling of the lung in asthma involves all anatomic compartments of the lung; specifically we hypothesized that α-SMA, a molecular marker of activated myofibroblasts [14] is increased in the lung parenchyma in asthma. To study this the density of are in the lung interstitium of patients who died of asthma, individuals with asthma who died of unrelated causes and individuals with no history of asthma who died of non-lung related disease were quantified and compared. This prospective pathologic/epidemiologic study was based on a historic cohort of asthmatics who died of their asthma in the Canadian Prairie Provinces of Manitoba, Saskatchewan and Alberta from 1992 to 1995. The study was initiated by Health Canada as a public health emergency in response to the very high death rate from asthma among young asthmatics [15]. It was preceded by a retrospective mortality study of 108 young asthmatics (age less than 33 years) who had died from their asthma in Alberta [16]. This study thus represents a unique cohort of asthmatics who died of asthma prior to contemporary standards of care.

We report that the pulmonary interstitium is profoundly altered by asthma and that the relationship between the lung parenchyma and airways in asthma needs to be revisited in light of these findings.

## Methods

### Study population

The study was based on autopsy materials collected and archived for the Canadian Prairie Provinces Asthma Study [13, 15] from November 1992 to October 1995 comprising Alberta, Saskatchewan and Manitoba. Cases were defined as subjects with asthma who died of asthma (fatal asthma, FA, n = 34); non-fatal asthma cases (NFA, n = 39) with a history of asthma who died of non-respiratory causes and non-asthma control cases (NAC, n = 35) who died with no history of asthma or other respiratory disease at death. Cases were obtained through the provincial Medical Examiners or Coroners’ Offices and local hospitals. Approval for the study was obtained from the University of Calgary Conjoint Ethics Committee. Written informed consent was obtained from next-of-kin for pathologic studies of lung tissues and access to medical examiner's, hospital and pharmacy records. A medical history, including details of asthma history (age of onset, asthma symptoms and severity, allergens, smoking history, asthma medications, hospitalizations due to asthma) and basic demographic information was also obtained from a questionnaire administered to the next-of-kin.

Inclusion criteria for this study were that subjects were life-time non-smokers with equal representation of males and females between groups. Eligible subjects from each group were selected randomly from those meeting these inclusion criteria. Only 7 subjects in any group met these conditions. The criteria for asthma diagnosis, comparison of groups by inclusion/exclusion and cause of death are given in supporting information (S1 Text, S2 Text and S1 Table).

### Lung sampling

A combined vascular/airway perfusion fixation was developed [17] in view of the marked mucous plugging of airways in the fatal asthma group [13]. The main bronchus and pulmonary artery were then perfused simultaneously with glutaraldehyde fixative (2.5% in 0.1 M phosphate buffer, pH 7.3, 350 mOsM with sucrose) at trans-pulmonary pressures of 20 and 40 cm H_2_O respectively, thus maintaining a capillary-alveolar pressure difference of 20 cm H_2_O.

Terminal bronchioles and surrounding lung parenchyma were sampled along the axial paths to the left anterior basal (LAB) and posterior basal (LPB) segments of the left lower lobe and from the apical segment (UAB) of the left upper lobe as previously described [13]. These samples were used for light microscopy, immuno-histochemistry and electron microscopy (EM). In addition portions of un-inflated peripheral lung from the right upper lobe were immersed in Zamboni’s fixative for EM.

### Histology and immunohistochemistry

Five micron thick sections of paraffin-embedded blocks were stained with hematoxylin and eosin and a modified Verhoeff Elastic Trichrome.

The sections for immunohistochemistry were deparaffinized, and 0.3% hydrogen peroxide solution applied for 35 minutes to inhibit endogenous peroxidase activity. Antigen retrieval was performed using a citrate solution for 45 min. The sections were incubated with the primary antibody for smooth muscle α-actin (1A4, Dako, Glostrup, Denmark). overnight at 4°C. A Novolink Polymer Detection System kit (Leica Biosystems Newcastle, UK) was used for the secondary antibodies (goat anti-mouse IgG).3,3diaminobenzidine (DAB) (Sigma Chemical Co., St Louis, MO, USA) was used as chromogen. The sections were counterstained with Harris hematoxylin. For negative controls, the primary antibody was replaced with PBS.

Additional sections were stained to demonstrate the dendritic nature of the myofibroblasts. The primary antibody was a mouse monoclonal IgG for α-SMA (MS 113 B1, Thermo Fischer Scientific, Walton, MA, USA). Biotinylated goat anti-mouse IgG was used as secondary antibody (BA-9200, Vector Laboratories, Burlingame, CA, USA.

### Feature identification and morphometry

A Carl Zeiss Axioplan light microscope, drawing tube and square lattice grid were used to determine the area fractions of selected features in the parenchyma using a point counting technique [13]. Parenchymal smooth muscle was measured by placing the grid randomly over sections stained for α-SMA. The field was accepted if it did not contain airways or blood vessels greater than 0.085mm in diameter. This measure was chosen as the cut off diameter for blood vessels in peripheral lung. Minor degrees of alveolar collapse were accepted. If any alveolar walls were opposed to each other, the field was rejected. The outer boundary was determined by a grid containing 120 points and magnification was set at 20X objective, with a distance between two points on the grid (Z) of 0.055 mm. The points lying over lung parenchyma containing tissue with and without actin were counted. The intersections of the grid lines with the basement membrane of the alveolar wall (BMint) were used to determine basement membrane length. Actin positive features were further classified by their location in alveolar duct, vascular and alveolar wall compartments. Three areas of distal lung were randomly selected and measured from the three segments. Inter-observer and intra-observer variability of the measured features were assessed on a sub-group of fifteen cases by two observers (SRB and FHYG). All measurements were made with the observers blinded to the case classification.

Area proportions of parenchymal features were calculated as: Area (μm^2^) = Z^2^.n, where Z is the distance between two points on the grid (magnification factor), and n is the number of points that land on the structure of interest. Alveolar surface length was calculated as: Luminal surface length (μm) = BMint * Z * π/4, where BMint is the number of grid intersections with the basement membrane, and Z is the magnification factor. The relative proportions of structures such as alveolar duct α-SMA, alveolar wall α-SMA, blood vessel α-SMA and total wall area were normalized to the luminal surface length as determined by: normalized area proportion (μm) = Area (μm^2^) / Luminal Surface Length (μm).

### Transmission electron microscopy (TEM)

Small (2 mm^3^) fragments of peripheral lung fixed in glutaraldehyde (inflated left lung) and/or Zamboni’s fixative (un-inflated right lung) were submitted for EM processing, sectioning and staining, as previously described [18]. Ultrathin sections stained with uranyl acetate and lead citrate were examined in a Hitachi 7650 transmission electron microscope. Smooth muscle and myofibroblasts were identified as previously described [18]. Ultra-structural features are considered to be the gold standard for the identification of myofibroblasts [18, 19].

### Data analysis

Coefficients of variation were used to assess inter-observer and intra-observer variation for each parameter. One-way analysis of variance was used for group (FA, NFA, and NAC) comparisons. The mean values for smooth muscle thickness in the upper lobe sections were compared to those in the lower lobes. The data used for interlobar comparisons were log(x+1) transformed before being analyzed by two way analysis of variance. Additionally, the data for alveolar wall smooth muscle and blood vessels as percent of total parenchyma were transformed using Box-Cox power transformation and square root transformation respectively. Analysis of variance was performed on Box-Cox transformed data for age. All transformations met the normality assumption. Tukey’s test was used for post-hoc analyses. A value of p < 0.05 was considered significant.

## Results

### Study population characteristics

Subject characteristics for case groups are shown in Table 1. For each group, there were four males and three females and all were non-smokers. Comparison of the study population with subjects not included in the study showed no significant differences for sex, use of B_2_ agonists or inhaled corticosteroids by group (Supporting Information S1 Text). Apart from smoking status the only difference noted was that the NFA subjects included in the study were significantly younger compared to the NFA subjects that were excluded (p = 0.002).Overall, the fatal asthma cases revealed a long history of persistent asthma, with greater use of β-agonists and oral corticosteroids than the NFA cases.

**Table 1:** Demographic characteristics of the study population: Fatal asthma (FA), non-fatal asthma (NFA) and non-asthma control (NAC) patients. (n = 7, all groups).

	FA	NFA	NAC
Sex (M/F)	4/3	4/3	4/3
Age mean years (range)	31.9, (18–59)	24.8, (18–29)	32.5, (18–50)
Asthma Duration (years mean ± SE)	13.6 ± 2.8	14.6 ± 4.0	N/A
Range (years)	6–26	2–31	
Treatment (% of subjects)			
*ß-agonists*	100	71.4	N/A
*Inhaled corticosteroids*	42.9	42.9	N/A
*Oral corticosteroid*	28.6	14.3	N/A

Abbreviations: F = female; M = male; N/A = Not Applicable

#### Parenchymal smooth muscle actin positive cells and their distribution

There were no significant differences in the calculated perimeters of alveolar wall and alveolar duct or total perimeter between the three groups. The intra-observer coefficient of variation ranged from 0.9–5.0% with a mean of 2.6% for estimates of alveolar duct and alveolar wall α-SMA + area. The inter-observer coefficient of variation was slightly higher with a mean of 5.4%.

**Fig 1:**
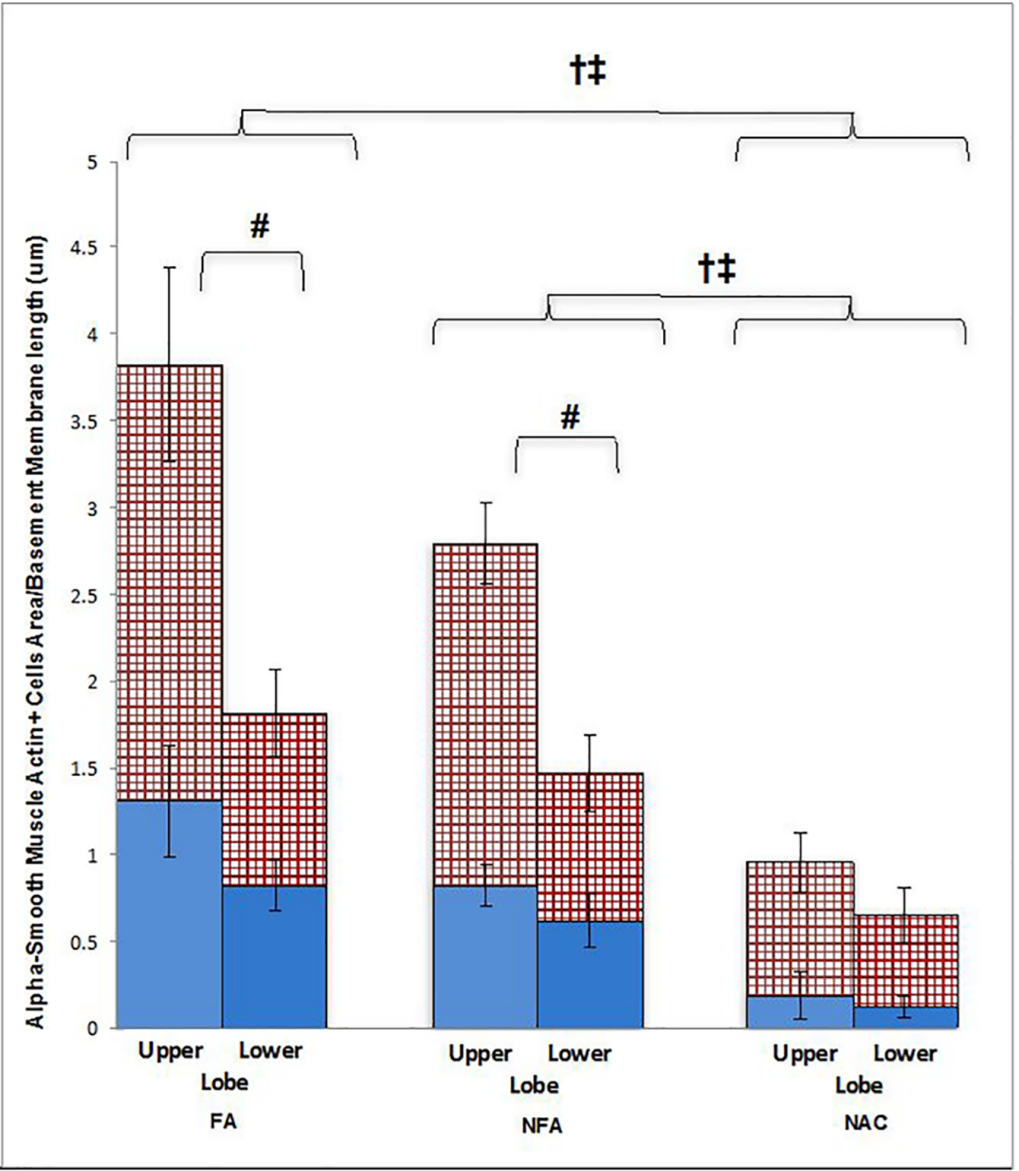
Area of α-SMA staining (normalized to basement membrane length) in alveolar ducts (blue) and alveolar walls (red hatched) of upper and lower lobes from cases of fatal asthma (FA) and non-fatal asthma (NFA), and from non-asthma control subjects (NAC). † p < 0.05 for alveolar ducts for both upper and lower lobes. ‡ p < 0.05 for alveolar walls for upper lobe only. # p < 0.01 upper vs lower lobes.

The α-SMA+ cells in the alveolar ducts and alveolar walls had similar staining characteristics and distribution in FA, NFA and NAC. α-SMA + cells were seen in the walls of respiratory bronchioles, arterioles, venules, alveolar ducts and in the alveolar parenchyma. When the area of α-SMA + staining was expressed as area per length of alveolar duct or alveolar wall, there were significant differences between FA and NFA compared with the NAC (Fig 1). However α-SMA staining was not significantly greater in FA compared with NFA (p = 0.38). Asthma cases (FA and NFA) had significantly more α-SMA + in their upper lobes compared with lower lobes (Fig 1). The difference between upper and lower lobes was not significant in the NAC group.

**Fig 2:**
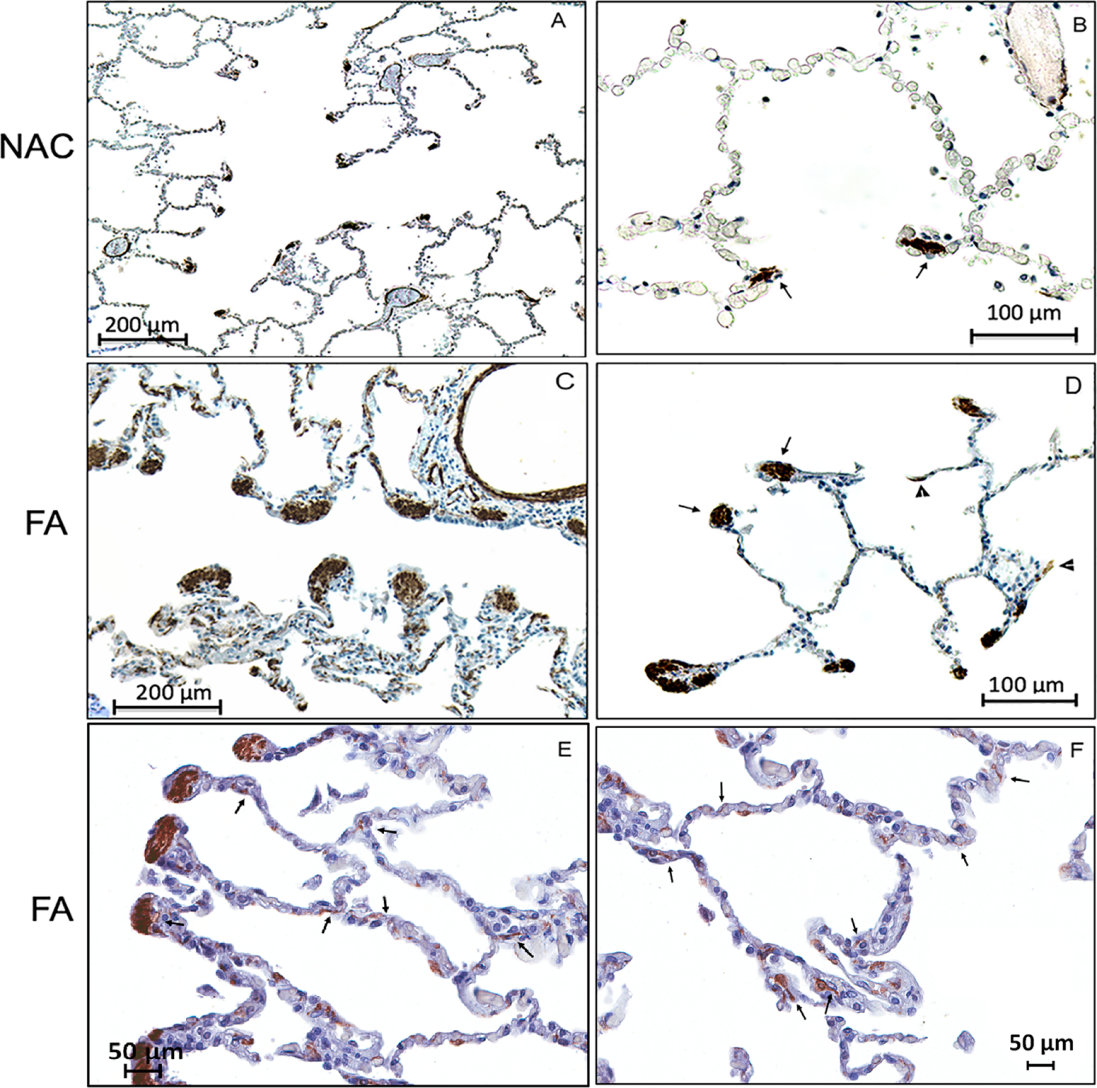
Examples of light micrographs from cases of non-asthma control (NAC) (A&B)and cases of fatal asthma (FA) (C,D,E&F) stained for alpha-smooth muscle actin. In the NAC case, the majority of the smooth muscle actin staining is seen in the alveolar duct tip (arrows) where it is concentrated with more subtle staining in the alveolar walls. By comparison, the FA case shows greater quantities of actin staining in the alveolar duct tip (arrow) with an even greater increase in alpha-smooth muscle actin staining in the alveolar walls (C-F arrowheads). At higher magnification (E,F) in addition to α-SMA + staining of the cell bodies there are α-SMA + cytoplasmic extensions of these cells into the adjacent interstitium providing a dendritic appearance (arrows).

The α-SMA + cells in the alveolar ducts and alveolar walls of asthma cases were larger than similarly staining cells in the NAC group and showed a dendritic morphology (Fig 2).

The relative areas (%) of α-SMA+ staining within the alveolar walls, alveolar ducts, and blood vessels are shown in Table 2. The proportion of α-SMA in the alveolar ducts was significantly greater in FA (p<0.001) and NFA (p = 0.02) compared with NAC.

**Table 2:** Area of alpha smooth muscle actin (α-SMA) staining in alveolar ducts, alveolar walls and blood vessels as a percentage of total parenchyma by group.

Group	Alveolar wall (%)	Alveolar ducts (%)	Blood vessels (%)
FA	14.7 ± 2.9†	9.4 ± 1.4*	6.7 ± 1.4
NFA	13.0 ± 1.2	7.1 ± 1.2‡	5.4 ± 1.4
NAC	7.5 ± 2.4	2.0 ± 0.8	3.9 ± 1.3

FA = fatal asthma, NFA = non-fatal asthma, NAC = non-asthma control

Values expressed as means ± standard error of the mean

† p = 0.048 FA vs NAC

*p = 0.0009 FA vs NAC

‡ p = 0.02 NFA vs NAC

Mean alveolar wall thickness was significantly greater in FA compared with NAC (p = 0.04) (Table 3). Alveolar wall thickness in NFA was increased but not significantly different from NAC (p = 0.08). The blood vessels were not significantly (p = 0.33) thicker in the asthma groups compared with NAC.

**Table 3:** Alveolar wall thickness normalized to basement membrane length (um) by group.

	FA	NFA	NAC
Alveolar wall thickness (μm) *	10.7 ± 0.7†	10.4 ± 0.4^‡^	8.6 ± 0.6

FA = fatal asthma, NFA = non-fatal asthma, NAC = non-asthma control

* Values expressed as means ± standard error of the mean

† p = 0.04 FA vs NAC

‡ p = 0.08 NFA vs NAC

### Electron microscopy

**Fig 3:**
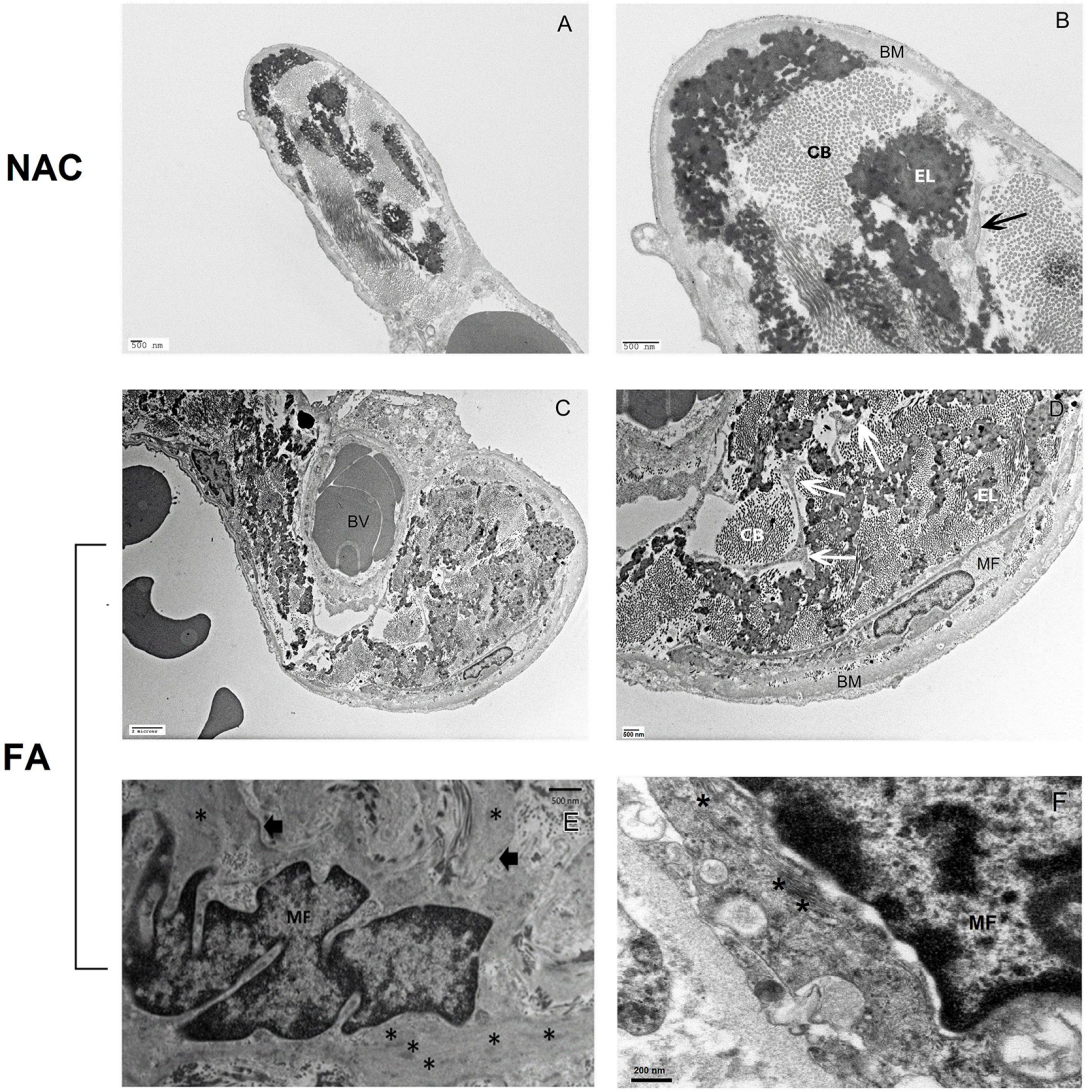
(A, B) Alveolar duct tip from a non-asthma control (NAC) subject showing the normal structure of the alveolar duct tip comprised of a thin layer of type I epithelium over a basement membrane (BM). These enclose bundles of elastin (EL) and collagen bundles (CB) Myofibroblasts were scarce in the NAC subjects but at higher magnification, extensions of their cytoplasm (arrow) can be seen (B). Panels C and D show an alveolar duct tip from a case of fatal asthma. The tip is composed of plates of elastin (EL), collagen (CB), a small blood vessel (BV), and a myofibroblast (MF). The myofibroblast shows cytoplasmic extensions (arrows) into and between the connective tissue bundles (D). The basement membrane (BM) is thickened. All components are increased compared with the non-asthma control subjects. At higher magnification (E, F), bundles of actin filaments (*), and fibronexi (solid arrows).are seen in panel E. The stellate/dendritic architecture of the myofibroblast in fatal asthma is seen in better detail with the cytoplasmic processes (arrows) extending between the collagen bundles and elastin plates (D).

Both glutaraldehyde and Zamboni’s fixatives provided clear differentiation of intracellular structures despite a moderate degree of autolysis. However Zamboni’s fixative was superior at preserving the actin fibres in the cytoplasm. Cells with ultra-structural features of myofibroblasts were identified in the alveolar ducts and in alveolar walls (Fig 3). Other cells containing actin filaments such as smooth muscle cells were identified in or around small blood vessels (Fig 3C). Myofibroblasts were identified by their spindle and/or stellate morphology, variable amounts of rough endoplasmic reticulum (RER), collagen secretion granules and fibronexi. The interstitial myofibroblasts lacked plasmalemmal caveolae. The structure of the alveolar tips consisted of bundles of collagen fibrils interspersed with plates of elastin. Myofibroblasts were located predominantly beneath Type 1 epithelial cell basement membrane with dendritic extensions into and between the collagen and elastic bundles (Fig 3B and 3D). The sub-epithelial membrane at the tips of the alveolar ducts was thickened in the asthma cases but not in NAC (Fig 3C and 3D) Overall the structural architecture was similar in all three groups (FA, NFA, and NAC) however FA showed increased size and numbers of myofibroblasts as well as increased amounts of collagen and elastin fibers.

## Discussion

This study has many limitations. First; the material was obtained as part of an epidemiologic study of fatal asthma at a time (mid 1990’s) that asthma mortality was high. As shown in Table 1 less than half (42.9%) the subjects were receiving inhaled corticosteroids, as, at that time, standard of care was primarily B2 agonists without inhaled corticosteroids as front line treatment. Second; the number of subjects that fulfilled the inclusion/exclusion was small, thus limiting the power of the study. This was due to a high number of cigarette smokers in the control population which had to be excluded. However we showed that subjects excluded from this study were similar in most respects to those that were included; see supporting information (S1 Table and Table A in S2 Text).

In this study we describe the density, and ultrastructure of α-SMA+ cells in the lung parenchyma of asthmatic and non-asthmatic control subjects. We show that asthmatics have significantly more α-SMA+ containing cells within their alveolar ducts and alveolar walls than non-asthmatic control subjects, and that these cells had the electron microscopic features of myofibroblasts [18]. These differences were greater in upper lobes compared with lower lobes in both asthma groups. There were no statistically significant differences between α-SMA+ staining cells in either alveolar ducts or walls between the fatal asthma cases and the non-fatal asthma cases. Increased α-SMA+ expression has been described in cells found in the airway submucosa [18], mucous glands [13], in airway elastic longitudinal bundles [20] and in the intimal layer of bronchial arteries [21] in asthma, indicating that the increase in myofilaments in asthmatic lungs may be a generic finding.

Whether this is an adaptive response to the biomechanical aspects of abnormal airway patho-physiology in asthma or a basic (genetic) expression of asthma is not apparent from the findings of this study or the published literature. The answer to this question has profound implications for treating severe asthma. Our discussion reviews the literature relevant to this question.

Weitoft *et al*. [3] previously reported an increase in alveolar interstitial myofibroblasts in patients with uncontrolled asthma compared with non-asthmatic subjects and patients with well-controlled asthma. They also showed increased parenchymal extracellular matrix including; collagen and proteoglycans in asthmatic patients. We confirmed an increase in α-SMA+ cells in the interstitium and also an overall increase in thickness of the alveolar interstitium in this study which are consistent with their findings. We extended these data to show that, in asthma, myofibroblasts are increased in both alveolar walls and alveolar ducts and that parenchymal SMA+ myofibroblasts are increased to a greater extent in the upper lobes than in lower lobes in both asthma groups. The latter effect does not appear to be an accentuation of a normal anatomy as no such effect was seen in the non-asthma controls. Alveolar ventilation and deposition of particles is greater in upper lobes compared with lower lobes [22]. Thus lobar differences observed in the present study may reflect greater antigenic loading and associated tissue response in the upper lobes.

The ultra-structural component of this study was designed to determine the phenotype of the cells containing actin fibres. This showed that the majority of interstitial cells in the locations indicated by SMA+ distribution had the ultra-structural characteristics of myofibroblasts. The EM features of myofibroblasts include crenated nuclear membranes, pinocytotic vesicles and scant bundles of microfilaments. In addition, EM in this study showed increased elastin, collagen bundles, and thickened basal lamina which was most marked over the alveolar duct tips in the asthmatic subjects. Alterations in myofibroblasts at the alveolar level have consequences for secretion of ECM molecules in asthma [3, 23]. Activated myofibroblasts are known to stimulate exaggerated production of extracellular matrix [14] after injury or mechanical stress stimulating growth factors to release ECM proteins [3, 24] that change the non-linear viscoelastic properties of the alveoli.

The mechanism(s) leading to the increase in α-SMA+ cells in the lung parenchyma are only partially understood. Inflammatory cells and mediators associated with asthma [2], acinar ventilation heterogeneity [9, 25, 26] or an adaptive response to ASM hypertrophy [27] may all be important. The origins of the increased ASM mass in asthmatic airways have also not yet been clarified [28]. Relationships between mast cells [29], eosinophil localization and ASM hypertrophy have been described in asthma [30]. Mast cells secrete chymase and activin A that regulate smooth muscle proliferation.[31]. Increased IgE and mast cells at the alveolar level have been observed in patients with uncontrolled asthma [4]. Influx of eosinophils and lymphocytes into the lung parenchyma have been described in nocturnal asthma [2]. Much more is known about actin regulation in airway smooth muscle than is known about actin regulation in the myofibroblast. The actin associated tyrosine kinase Abl is a key regulatory protein of actin dynamics, controlling critical pathways for force generation, cell proliferation, cell adhesion and migration of airway smooth muscle cells [32, 33]. Furthermore it can be inhibited by the drug Imatinib, an inhibitor of the tyrosine kinase activity of KIT, which decreases mast cell function and improves lung function in severe asthma [34]. Whether this approach would be useful or appropriate for treating asthmatic parenchymal remodelling requires further study and consideration.

Lung parenchymal mechanics is profoundly altered in asthma [9, 26, 35]. To constrict airways, airway smooth muscle must overcome loads imposed by the lung parenchyma. The alveolar attachments to the airway walls in combination with pleural pressure create an external load against which airway smooth muscle contracts. Contraction of airway smooth muscle in asthma can overcome the internal loads of the airway wall and result in excessive airway narrowing [36]. This is compounded by the amplifying effects of increased wall thickness on normal muscle shortening [37].

An increase in parenchymal myofibroblasts in asthma might act as a protective mechanism by increasing recoil tension and preventing collapse of small airways during changes in lung volume. This effect has been observed experimentally in dogs [25]. However studies of lung elastic recoil and compliance in asthma are not consistent, being variably reported to be normal [38], increased [39], decreased [40, 41] or reversible [41]. Increased α-SMA in the lung parenchyma might increase lung hysteresis, which usually contributes little to total lung hysteresis at low breathing frequencies but predominates at high breathing frequencies [42]. Increased lung hysteresis, relative to airway wall hysteresis should augment bronchodilation, especially with deep breaths [43]. However in asthma, the physiologic effect of a deep breath is usually diminished, enhancing bronchoconstriction [44]. This suggests that the effects of the increase in smooth muscle in the airway wall are greater than those in the lung parenchyma. Colebatch *et al*. [45] showed that in asthma, airway conductance was reduced relative to lung elastic recoil and Brown *et al*. [27] found that with induced airway smooth muscle constriction parenchymal attachments could no longer distend the airways at increased lung volumes. A loss of normal alveolar attachments in fatal asthma might contribute to the impairment of the transmission of forces from parenchyma to airways [46].

It is also not understood if parenchymal smooth muscle responds to stimuli in the same manner as airways. The innervation of small airways is sparse compared with large airways and almost non-existent in the pulmonary parenchyma [47]. In addition, receptor distribution is asymmetric in the mammalian lung [48]. The significance of regional differences in receptor distribution is not known but may have important implications when targeting drugs to the lung.

In summary, there is approximately a three-fold increase of parenchymal α-SMA+ cells in asthmatics dying of asthma and also in asthmatics dying from unrelated causes, compared to non-asthmatics. We also show a significant increase in α-SMA in the upper lobes compared with the lower lobes in asthmatic subjects but not in the non-asthmatic subjects. The significance of the increase in interstitial myofibroblasts in alveolar walls and ducts in asthmatic lungs is not entirely clear but our findings are critical for an informed understanding of the relationship between airway and parenchymal mechanics. A better understanding the pharmacology and patho-physiology of these α-SMA+ interstitial cells has important implications for asthma management.

## Supporting information

S1 Text Diagnosis of asthma. (DOCX)

S2 Text Characteristics of the study groups by included and excluded subjects. (DOCX)

S1 Table Cause of death and age for study subjects by group. (DOCX)

S1 Dataset Raw data used for the study. (XLSX)
