# Supplementary material for: Myofibroblasts are increased in the lung parenchyma in asthma
Source: PLoS One. 2017 Aug 7;12(8):e0182378. doi: 10.1371/journal.pone.0182378 (PMC5546673; doi:10.1371/journal.pone.0182378)
Supplement: S1 Text — (DOCX) [file pone.0182378.s001.docx]

**S1 Text 1**

**Diagnosis of asthma**

A total of 108 subjects with complete autopsies were enrolled into the study, of these 34 were FA, 39 were NFA and 35 were NAC. From this group we selected seven life-time non-smokers for this study with equal male and female distribution between groups. The diagnosis of asthma was made on clinical and pathologic criteria. Clinical criteria included a reported diagnosis of asthma by a doctor and the presence of episodic symptoms of wheeze, chest tightness, cough or shortness of breath. The severity of asthma was assessed based on frequency of symptoms, treatment requirements and the effect of asthma on school or work attendance [1]. The pathologic criteria were grade (0-3) of airway smooth muscle thickening, lymphocytic and eosinophilic inflammation, thickening of the laminar reticularis (basement membrane) and mucous cell hyperplasia. Death from asthma was defined as histological evidence of severe asthma as defined by the above criteria, together with evidence of asphyxia; airway mucous plugging, lung hyperinflation and/or collapse) and forensic attributes of petechial haemorrhages of the serosal cavities. Other potential causes of death were eliminated following examination of toxicology reports and findings at autopsy.

**Reference (1)**

Global Initiative for Asthma. Global Strategy for Asthma Management and Prevention, 2017. Available from: http://ginasthma.org/ [Cited July.6^th^ 2017].
