# Supplementary material for: Myofibroblasts are increased in the lung parenchyma in asthma
Source: PLoS One. 2017 Aug 7;12(8):e0182378. doi: 10.1371/journal.pone.0182378 (PMC5546673; doi:10.1371/journal.pone.0182378)
Supplement: S2 Text — (DOCX) [file pone.0182378.s002.docx]

**S2 Text**

**Characteristics of the study groups by included and excluded subjects**

Subjects meeting the inclusion criteria (life-time non-smokers with equal male and female subjects in each group) were randomly selected from eligible subjects. Exclusion of smokers allowed study of the effects of asthma on lung parenchyma without the confounding effects of smoking. To determine if those selected for the study were representative of all subjects in the same group we undertook the following analysis.

Data analysis

Pearson's Chi-squared test with Yates' continuity correction was performed to determine if there were any differences in subject characteristics in terms of gender, ß-agonist use, inhaled steroid use and smoking status between the included and the excluded groups.

In order to determine if there were any differences in age and years with asthma between the included and excluded groups, Two-tailed T test was carried out. The age data for the non-fatal asthma group was log transformed in order to meet the assumption of normality. A p value < 0.05 was considered significant. Table 1 shows summary characteristics of the study population and the excluded population, including the p values following analyses. All of the statistical analyses were done in R Statistical Software Version 3.2.0 GUI 1.65.

There were significant differences in the smoking status between the included and the excluded groups belonging to fatal asthma (p=0.02), non-fatal asthma (p=0.001) and non-asthmatic controls (p=0.003). The only other difference was that the subjects included in the non-fatal asthma group were significantly younger compared to those excluded (p= 0.002).

Table A. Characteristics of the included and the excluded study groups

|  | **Included** | **Excluded** | **p-value** |
| --- | --- | --- | --- |
| **Fatal Asthma**  Sex (M/F) | (n=7)  4/3 | (n=27)  17/10 | 1 |
| Age (years) ^‡^ | 31.9 ± 6.7 | 32.1 ± 2.5 | 0.87 |
| Years with Asthma ^‡^ | 13.57 ± 2.8 | 19.87 ± 2.4 | 0.21 |
| ß-agonist use (% of subjects):  Everyday  Occasionally | 85.7  14.3 | 88.5  11.5 | 1 |
| Inhaled Steroid use (% of subjects): Everyday  Occasionally  None | 28.6  14.3  0 | 57.1  28.6  14.3 | 0.78 |
| Smoker (Y/N) | 0/7 | 15/11 | 0.02* |
| **Non-Fatal Asthma**  Sex (M/F) | (n=7)  4/3 | (n=32)  14/18 | 0.83 |
| Age (years) ^‡^ | 24.8 ± 1.9 | 40.2 ± 2.1 | 0.002* |
| Years with Asthma ^‡^ | 14.6 ± 4.0 | 18.4 ± 2.4 | 0.48 |
| ß-agonist use (% of subjects):  Everyday  Occasionally  None | 42.9  42.9  0 | 39.3  50.0  10.7 | 0.66 |
| Inhaled Steroid use (% of subjects): Everyday  Occasionally  None | 40.0  20.0  40.0 | 44.4  27.8  27.8 | 0.86 |
| Smoker (Y/N) | 0/7 | 23/8 | 0.001* |
| **Non Asthmatic Control**  Sex (M/F) | (n=7)  4/3 | (n=28)  18/10 | 1 |
| Age (years) ^‡^ | 32.5 ± 4.2 | 43.2 ± 2.5 | 0.05 |
| Smoker (Y/N) | 1/6 | 22/5 | 0.003* |

^‡^ Data expressed as mean and ± standard error.

*Significant (p value < 0.05)
