# Supplementary material for: Myofibroblasts are increased in the lung parenchyma in asthma
Source: PLoS One. 2017 Aug 7;12(8):e0182378. doi: 10.1371/journal.pone.0182378 (PMC5546673; doi:10.1371/journal.pone.0182378)
Supplement: S1 Table — (DOCX) [file pone.0182378.s003.docx]

**S1 Table**

**Cause of death and age for study subjects by group.**

| **Group** | **Case** | **Age** | **Cause of death** |
| --- | --- | --- | --- |
| FA | H253 | 55 | Asthma |
| FA | H248 | 59 | Asthma |
| FA | H296 | 18 | Asthma |
| FA | H297 | 18 | Asthma |
| FA | H316 | 24 | Asthma |
| FA | H324 | 20 | Asthma |
| FA | H245 | 46 | Asthma |
| FA | H266 | 16 | Asthma |
| FA | H323 | 26 | Asthma |
| FA | H260 | 29 | Asthma |
| NFA | H234 | 29 | Intraventricular hemorrhage |
| NFA | H235 | 33 | Drug toxicity, obesity |
| NFA | H236 | 21 | Acute ethanol toxicity |
| NFA | H269 | 23 | Asphyxia, suffocation in avalanche |
| NFA | H314 | 25 | Morbid obesity, enlargement of heart and liver |
| NFA | H334 | 18 | Diabetic ketoacidosis |
| NFA | H279 | 25 | Congenital heart disease |
| NFA | H308 | 45 | Multiple drug toxicity, COPD, obesity |
| NFA | H259 | 46 | Pulmonary congestion, edema, chronic heart disease |
| NFA | H329 | 22 | Epilepsy |
| NFA | H306 | 35 | Acute myocardial infarction, atherosclerosis |
| NAC | H338 | 33 | Pneumonia, neural and musculoskeletal dev. disorder |
| NAC | H271 | 40 | Hepatocellular carcinoma, Marfan's syndrome |
| NAC | H267 | 42 | Primary dissecting aneurysm of the left coronary artery |
| NAC | H339 | 41 | Renal transplant rejection, CMV enteritis, pneumonia |
| NAC | H288 | 50 | Leukemia |
| NAC | H330 | 21 | Motor vehicle accident |
| NAC | H331 | 18 | Brainstem tumor, acute bronchopneumonia |
| NAC | H332 | 33 | Subarachnoid hemorrhage |
| NAC | H340 | 29 | Seizure disorder |
| NAC | H343 | 31 | Self-inflicted gunshot wound to the head |

FA = Fatal asthma, NFA = Non-fatal asthma, NAC = Non-asthma control
